# Implementing an exclusive human milk diet for preterm infants: real-world experience in diverse NICUs

**DOI:** 10.1186/s12887-023-04047-5

**Published:** 2023-05-12

**Authors:** Jonathan R. Swanson, Amy Becker, Jenny Fox, Michael Horgan, Russell Moores, John Pardalos, Joaquim Pinheiro, Dan Stewart, Tonya Robinson

**Affiliations:** 1 University of Virginia Children’s Hospital, Charlottesville, VA USA; 2 Shady Grove Medical Center, Baltimore, MD USA; 3 Children’s Hospital of Richmond at Virginia Commonwealth University, Richmond, VA USA; 4 Division of Neonatal Medicine, Albany Medical Center, Bernard & Millie Duker Children’s Hospital, Albany, NY USA; 5 University of Missouri Health Care-Columbia, Columbia, MO USA; 6 Albany Medical Center, Bernard & Millie Duker Children’s Hospital, Albany, NY USA; 7 Norton Children’s Hospital and University of Louisville School of Medicine, Louisville, KY USA; 8 University of Louisville Hospital, Louisville, KY USA

**Keywords:** Exclusive human milk diet, Human milk fortifier, Preterm birth, Necrotizing enterocolitis, Growth, NICU, Cost, Implementation, Public policy, Advocacy

## Abstract

**Background:**

Human milk–based human milk fortifier (HMB-HMF) makes it possible to provide an exclusive human milk diet (EHMD) to very low birth weight (VLBW) infants in neonatal intensive care units (NICUs). Before the introduction of HMB-HMF in 2006, NICUs relied on bovine milk–based human milk fortifiers (BMB-HMFs) when mother's own milk (MOM) or pasteurized donor human milk (PDHM) could not provide adequate nutrition. Despite evidence supporting the clinical benefits of an EHMD (such as reducing the frequency of morbidities), barriers prevent its widespread adoption, including limited health economics and outcomes data, cost concerns, and lack of standardized feeding guidelines.

**Methods:**

Nine experts from seven institutions gathered for a virtual roundtable discussion in October 2020 to discuss the benefits and challenges to implementing an EHMD program in the NICU environment. Each center provided a review of the process of starting their program and also presented data on various neonatal and financial metrics associated with the program. Data gathered were either from their own Vermont Oxford Network outcomes or an institutional clinical database. As each center utilizes their EHMD program in slightly different populations and over different time periods, data presented was center-specific. After all presentations, the experts discussed issues within the field of neonatology that need to be addressed with regards to the utilization of an EHMD in the NICU population.

**Results:**

Implementation of an EHMD program faces many barriers, no matter the NICU size, patient population or geographic location. Successful implementation requires a team approach (including finance and IT support) with a NICU champion. Having pre-specified target populations as well as data tracking is also helpful. Real-world experiences of NICUs with established EHMD programs show reductions in comorbidities, regardless of the institution’s size or level of care. EHMD programs also proved to be cost effective. For the NICUs that had necrotizing enterocolitis (NEC) data available, EHMD programs resulted in either a decrease or change in total (medical + surgical) NEC rate and reductions in surgical NEC. Institutions that provided cost and complications data all reported a substantial cost avoidance after EHMD implementation, ranging between $515,113 and $3,369,515 annually per institution.

**Conclusions:**

The data provided support the initiation of EHMD programs in NICUs for very preterm infants, but there are still methodologic issues to be addressed so that guidelines can be created and all NICUs, regardless of size, can provide standardized care that benefits VLBW infants.

## Background

Over the past decade, there has been an enhanced focus on nutrition to improve short- and long-term outcomes for very low birth weight (VLBW; ≤ 1500 g) infants in neonatal intensive care units (NICUs). Multiple studies have demonstrated the benefits of human milk as the primary form of enteral nutrition, with data showing reduced morbidity and mortality in the VLBW infant; however, these studies also provide evidence that mother's own milk (MOM) or pasteurized donor human milk (PDHM) alone lacks the energy and protein required to promote adequate growth and development in the VLBW preterm infant [1–6].

In 2012, the American Academy of Pediatrics (AAP) recommended that all preterm infants receive human milk instead of formula when possible, and this guidance was reaffirmed in 2022 [7, 8]. The AAP also recommends fortification of human milk as the standard of care in VLBW infants to ensure optimal nutrient intake; if MOM is unavailable or its use contraindicated, PDHM should be used. These recommendations support the use of an exclusive human milk diet (EHMD)—completely free of any bovine milk–derived components—in this vulnerable patient population.

### The benefits of EHMD

An EHMD consists of MOM, PDHM, and fortification, when necessary, with human milk–based human milk fortifier (HMB-HMF) and not bovine milk–based human milk fortifier (BMB-HMF). Reported benefits of an EHMD include a healthier growth velocity [9], appropriate neurodevelopment [9, 10], decreased rates of comorbidities [1, 3–6], decreased rates of disability [11], decreased rates of mortality [1, 5], and development of a healthy immune system [12–15]. Infants fed an EHMD tolerate enteral feeding better and advance to full enteral feeds more quickly [3]. In addition, EHMD administration is associated with reductions in specific comorbidities commonly seen in VLBW infants, including retinopathy of prematurity (ROP), bronchopulmonary dysplasia (BPD), necrotizing enterocolitis (NEC), and late-onset sepsis, thereby reducing costs incurred by medical interventions and longer length of stay (LOS) in the NICU [3, 11, 16–20].

### Barriers to EHMD administration

Despite the benefits of an EHMD, there are some significant challenges that prevent this feeding program from being more universally implemented in the United States. For instance, the cost of an EHMD program—including staffing, procurement of space, specialized equipment, and purchase of PDHM/HMB-HMF—is one of the primary barriers to adoption [11]. Another major obstacle is the lack of standard EHMD feeding protocols suitable for a variety of NICUs. Institutions have had to formulate their own EHMD workflows and eligibility criteria, which are often based on factors specific to each NICU, such as number of beds, patient population, funding landscape, preexisting comorbidity burden, and staff availability. This also makes it difficult to compare post-EHMD costs and outcomes across institutions.

Despite evidence from clinical trials supporting the benefits of EHMD for VLBW preterm infants in the NICU, there is a lack of robust real-world clinical data on the efficacy and cost-effectiveness of this approach. Insights from real-world EHMD implementation experiences of seven diverse NICUs are presented in the hope of beginning to address this issue.

## Methods

A virtual roundtable was held in October 2020. Hospitals that were known to utilize an EHMD were asked by one author (JRS) to participate. In addition to the chair, eight neonatologists from six diverse (size of NICU, patient population, geographic location) NICUs agreed to participate. Prior to the roundtable, participants were asked to develop a presentation on the process of obtaining institutional support for using an EHMD in their NICU as well as clinical, and if available, financial metrics since starting their program. As each NICU utilized an EHMD in different populations and also over different time periods, specific data points were not required. As most hospitals are part of the Vermont Oxford Network (VON), the chair asked that the 2020 VON definition for clinical variables be utilized for presentation of data. Participating hospitals utilized their VON data or institutional specific clinical databases. IRB approval for research or quality improvement was determined by each participating institution.

During the roundtable, the presenting institution would provide qualitative issues surrounding the implementation of an EHMD program and subsequently provided an overview of metrics available. After all seven hospitals presented, an open discussion was held on issues preventing further dissemination of EHMD programs across neonatology. The presentations and discussions during this roundtable led to the creation of this manuscript describing real-world experiences, challenges and future directions regarding EHMD programs.

### Overview of the NICUs providing real-world experiences with EHMD

Table 1 summarizes the NICU characteristics of the participating institutions providing real-world data. Average annual admissions ranged from 230 for a small, level 3 NICU serving military personnel and their families to 1450 for a large, level 4 NICU at a major academic center. Average annual VLBW births at participating institutions also ranged widely, from 16 to 250. The number of years since EHMD implementation ranged from 2 to 12. Funding and reimbursement for EHMD products varied, with most institutions reporting at least some reimbursement, although reimbursement for PDHM appears to be more common than for HMB-HMF (data not shown). Payor mix (self-pay, private insurance, Medicaid) also differed between institutions, with the proportion of Medicaid patients ranging from 0 to 75%.

**Table 1:** Participating NICU characteristics

	**NICU Type**	**NICU Admissions (average per year)**	**Average Daily Census**	**VLBW Births (average per year)**	**Proportion of Medicaid** **Patients**	**Years Since EHMD Implementation**
University of Virginia Charlottesville, VA	Level 4	700	53	120	50%	6
Children’s Hospital of Richmond Richmond, VA	Level 4	450	35	80–100	29%–35%	4
Albany Medical Center Albany, NY	Level 4	800	48	140	57%	2
University of Missouri Children’s Hospital Columbia, MO	Level 3	600–700	45	80–100	55%	5
Madigan Army Medical Center Tacoma, WA	Level 3	230	9	16–20	0%^a^	3
University of Louisville Louisville, KY	Level 3	445	21	60	75%	9
Norton Children's Hospital Louisville, KY	Level 4	1180–1450	80	150–250	60%	10

^a^Patients at this hospital are covered by Tricare health insurance for United States military personnel and their dependents

### EHMD programs: benefits, challenges, and real-world experiences

#### Creating the EHMD Program in a NICU

Implementing and maintaining an EHMD program is a complex process requiring a multidisciplinary team. A physician champion and/or dedicated committee can provide critical guidance and motivation, from formulating a proposal for financial support to execution of the EHMD protocol, monitoring of clinical outcomes, and ongoing cost–benefit analyses.

Education on the benefits of an EHMD and a clear understanding of its implementation are necessary for staff buy-in across a range of team member functions. Clear workflows for milk mixing and administration should be defined in advance, with documentation available for reference. New staff, such as milk technicians, may be required and existing staff members may have to assume new responsibilities. Ordering, storage, and preparation of EHMD products must all be integrated into the existing NICU workflow. Appropriate information technology infrastructure and support is also needed for tracking use of product (e.g., though the EMR) and recording/analyzing outcomes.

The hospital administration may also require periodic cost updates as part of an ongoing review of funding, which requires active monitoring of expenditures, along with clinical outcomes. Given the shifting regulatory and reimbursement landscape, the assistance of finance experts may be critical to the sustainability of EHMD programs.

##### Real-world experiences

Most of the EHMD programs at the 7 institutions contributing to this report began with a proposal outlining the cost of an EHMD and potential cost avoidance due to reduced morbidity and LOS. Participation of finance and supply staff in these early discussions and after EHMD implementation was important, as costs and outcomes may change over time, particularly with changes in the patient population, reimbursement regulations, and clinical practices.

All institutions reported changes in staffing, with most requiring enhanced lactation and dietician support, at least during initial EHMD implementation. Several set up separate rooms/areas for breastmilk fortification, which was done by a dedicated team of nurses, patient care technicians or registered dieticians. There was general consistency in the ratios of bedside registered nurses (RNs), lactation support personnel, and dieticians among the NICUs.

#### EHMD eligibility criteria

There are currently no guidelines from medical associations, such as the American Academy of Pediatrics, or from the National Institutes of Health as to the minimum weight and age at which VLBW infants in the NICU should begin EHMD.

##### Real-world experiences

Perhaps the largest variability in EHMD implementation strategies across institutions was seen in the eligibility criteria (Table 2). The maximum birth weight for EHMD administration ranged between 1000 and 1500 g among the NICUs, which was largely due to differences in local funding constraints. Three of the seven NICUs included a postmenstrual age (PMA) cutoff in their EHMD eligibility criteria, 1 at 28 weeks, 1 at 29 weeks, and 1 at 32 weeks. One NICU received permission from hospital administrators after program implementation to increase the birth weight cutoff from 1000 to 1250 g, based on improved outcomes and cost avoidance data. All institutions required parental consent, either verbal or written, for administration of PDHM.

**Table 2:** EHMD implementation parameters of participating NICUs

	**Fortification Goal (kcal/oz)**	**Feeding Volume at Fortification (mL/kg/oz)**	**EHMD Inclusion Criteria**	**Criteria to Transition off EHMD**
University of Virginia	26–28	80	≤ 1250 g; < 28 weeks' PMA	34 weeks' PMA
Children’s Hospital of Richmond	26–28	80	≤ 1250 g	34 weeks' PMA
Albany Medical Center	26–32	60	≤ 1500 g	34 weeks' PMA
University of Missouri Children’s Hospital	26–28	80	< 1000 g; < 29 weeks' PMA	4 weeks after 20 mL/kg/d feeds
Madigan Army Medical Center	26–28	60	< 1500 g; < 32 weeks' PMA	> 1800 g; 34 weeks' PMA
University of Louisville	26–28	60	≤ 1000 g	≥ 33 weeks' PMA
Norton Children's Hospital	26–28	60	≤ 1000 g	≥ 32 weeks' PMA

#### Developing feeding protocols

There are currently no guidelines regarding EHMD feeding in the NICU, but use of standardized feeding protocols developed by individual institutions has produced improved outcomes, including lower NEC rates, faster advancement to full enteral feeds, fewer TPN/central line days, fewer sepsis evaluations, and improved growth rates [21–27]. In addition, feeding guidelines for at-risk neonates improve outcomes by reducing variation between NICUs. Published evidence-based feeding guidelines representing a consensus among experts in the field could increase confidence in the benefits of an EHMD among hospital administrators/NICU staff and potentially simplify uptake of this approach.

##### Real-world experiences

EHMD implementation required changes to existing feeding protocols or creation of new feeding protocols at all seven NICUs. Clear workflows for milk mixing and administration were defined in advance, and documentation of these procedures was made available to NICU staff. Adjustments to feeding protocols at individual NICUs during and after initial EHMD implementation were based on review of the literature and assessment of outcomes.

EHMD implementation parameters were set individually by each participating institution (Table 2). Although protocols differed, feeding volume at initiation of fortification, fortification goals, and criteria for transitioning infants off the EHMD were similar. Three NICUs initiate fortification when the infant reaches an enteral feeding volume of 60 mL/kg/d, whereas the other four initiate fortification at a feeding volume of 80 mL/kg/d. Six of seven NICUs set a fortification goal of 26–28 kcal/oz, whereas the seventh has a goal of 26–32 kcal/oz. Most participating NICUs continue HMB-HMF fortification until infants reach 32- or 34-weeks PMA; however, 1 adds a current weight requirement of > 1800 g, whereas another transitions infants off the EHMD 4 weeks after achieving 20 mL/kg/d feeds. Again, guidelines are needed to help simplify feeding protocols and make it easier to institute an EHMD program.

#### Decrease in comorbidity rates with EHMD

Careful assessment of EHMD-related outcomes is vital not only to justify initial and ongoing funding but also to refine feeding practices to maximize VLBW infant growth and health. As a major driver of per-patient NICU costs and, potentially, mortality or long-term complications, a reduction in NEC is a good benchmark for EHMD efficacy that also makes a persuasive argument for funding [11, 17, 28]. Other comorbidities that may be affected by NICU diet, including BPD, ROP, late-onset sepsis, central line-associated blood stream infection (CLABSI), and neurodevelopmental impairment, should also be monitored. Nutritional parameters—including days until full feeds, feeding intolerance, and need for additional supplementation with electrolytes or lipids—provide insight into EHMD efficacy. Growth parameters of patients receiving an EHMD should be followed in the NICU and, ideally, after discharge. Long-term monitoring should include an evaluation of each patient throughout childhood for developmental issues and into adulthood for the occurrence of adult-onset diseases (e.g., diabetes and hypertension).

##### Real-world experiences

**Fig. 1:**
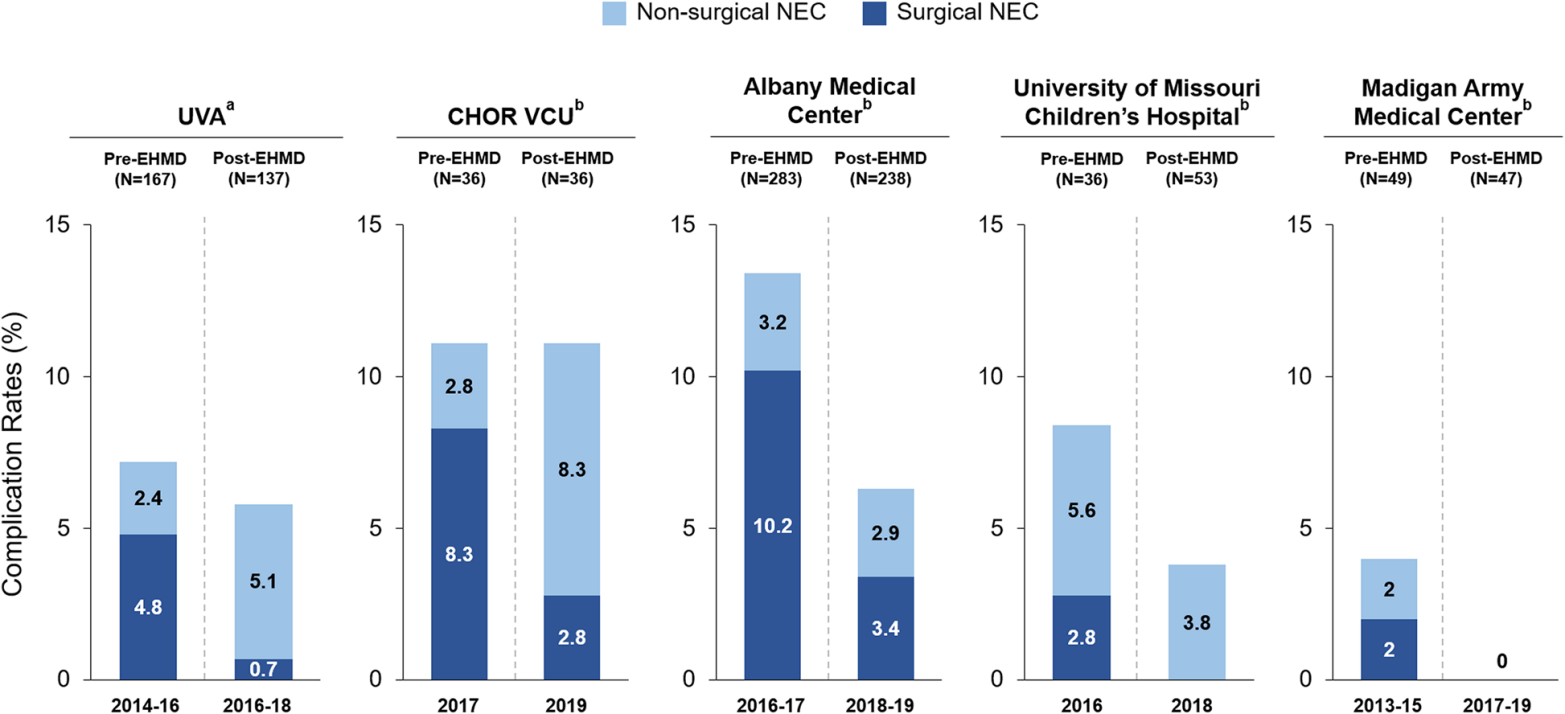
Nonsurgical and surgical NEC rates before and after EHMD implementation. Number of patients and years comprising each cohort are shown

Reducing NEC rates is often cited as a primary motivation for initiating an EHMD program; thus, it is critical that the incidence of NEC be closely monitored, pre- and post-EHMD implementation. Of the 5 institutions providing NEC data, 4 reported reductions in total NEC incidence (non-surgical + surgical), and 1 reported no change. These reductions were largely driven by decreases in surgical NEC at all 5 institutions, ranging from 66 to 100%. Two institutions reported increases in the incidence of non-surgical NEC (while 3 showed reductions), but these increases were offset by reductions in surgical NEC cases. The timespans for the reported NEC data varied by institution and are specified in Fig. 1.

**Fig. 2:**
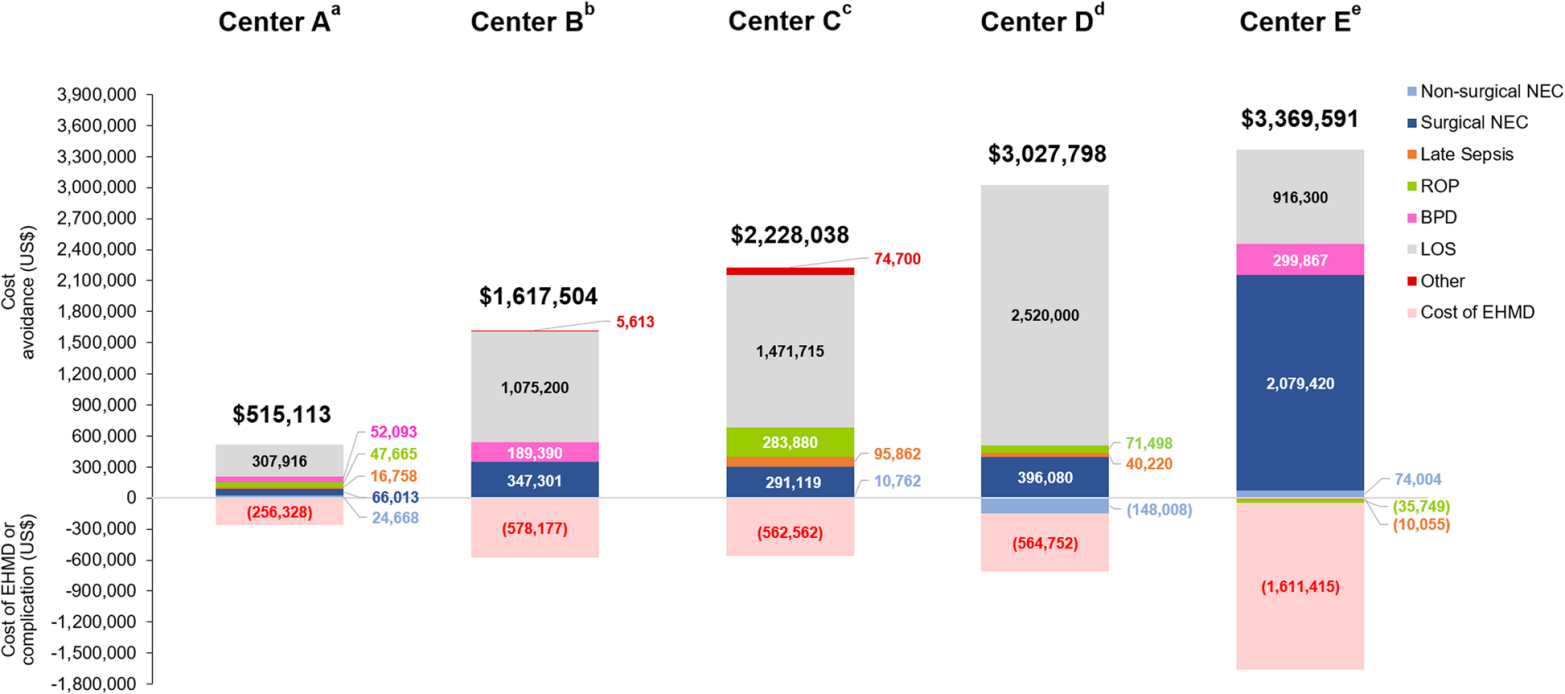
Annualized costs and cost avoidance with EHMD use by center (anonymized). Total and itemized cost avoidance are shown. Information on other costs was not uniformly available at all centers

Reductions in other complications of prematurity that may be affected by NICU diet—including late-onset sepsis, BPD, severe ROP, and central line-associated-blood stream infection (CLABSI)—were also generally observed across participating institutions after EHMD implementation (data not shown, but are represented in the cost outcomes in Fig. 2).

#### Costs and savings of an EHMD program

It should be emphasized that the cost of an EHMD—approximately $12,500 for a 90-day NICU hospitalization—represents only a fraction of the usual cost of care for a VLBW infant (90 days: $693,00 to $774,000 depending on level of care) [11, 29]. Any reduction in LOS can have a sizeable impact on total cost expenditure compared with the investment in EHMD.

##### Real-world experiences

Five of the participating institutions reported data on the costs associated with EHMD implementation (Fig. 2). Per institutional guidelines, these data were anonymized. Annual cost avoidance due to EHMD implementation ranged between $515,000 and $3,370,000 per year. The wide range reflects the differences in the number of VLBW infants admitted to each NICU, variations in feeding protocols, and disparities in reimbursement. EHMD implementation at all participating institutions was, at minimum, cost neutral, with the potential for significant savings due to reductions in morbidity and LOS.

### Making the case for EHMD adoption to hospital administration

The AAP affirmed in their 2012 and 2022 “Breastfeeding and the Use of Human Milk” policy statements that infants should be exclusively breast fed for the first 6 months of life; however, MOM sometimes is unavailable or not available in sufficient quantity to meet an infant’s nutritional needs [7, 8]. For this reason, PDHM and HMB-HMF are commonly used to support adequate growth in VLBW infants. Unfortunately, the cost of PDHM and human milk–based products is a major issue for most hospitals exploring EHMD implementation. Currently, only California, Connecticut, Illinois, Kansas, Kentucky, Louisiana, Missouri, New Jersey, New York, Ohio, Oregon, Pennsylvania, Texas, and Utah, and Washington, DC, have laws that require Medicaid programs to reimburse PDHM costs [30]. In addition, Florida, Maine, Massachusetts, Nevada, Oklahoma, South Carolina, and Virginia have had bills in recent legislative sessions that will mandate coverage for PDHM, if passed. Tricare (U.S. Defense Health Agency, Falls Church, VA) health insurance for United States military personnel and their dependents covers banked PDHM for certain medical conditions. The neonatology community must support ongoing and future efforts to require insurance companies and Medicaid to reimburse hospitals for the use of this “medicine” in our most vulnerable patients.

Advocacy efforts must also include education of lawmakers and regulatory authorities on the short- and long-term benefits of an EHMD for VLBW infants, particularly the cost savings realized by preventing comorbidities of preterm birth [30].

#### Real-world experience

Unfortunately, it is the experts’ experiences that EHMD adoption is hindered by the reality that its implementation is a complex process. Practical lessons learned over time that may facilitate EHMD implementation include building a case for starting an EHMD program, the importance of standardized feeding protocols, building an EHMD team, identifying which outcomes to monitor, and the need for advocacy in the neonatology community.

Each institution has its own decision-making process for determining whether to implement a new therapy. Institutions also vary in terms of the availability of reimbursement for EHMD products, need for new staff to implement an EHMD, financial/insurance status of patients, and existing comorbidity burden. If comorbidity rates are already low, that may affect the ability to make a compelling argument for EHMD funding. When developing a proposal for an EHMD program, the implementation team must educate the hospital administration on the benefits of an EHMD beyond reductions in comorbidities, including improved growth, shorter LOS, and better long-term outcomes. It should be emphasized that the cost of an EHMD—approximately $12,500 for a 90-day NICU hospitalization—represents only a fraction of the usual cost of care for a VLBW infant [11]. Cost-savings data from other institutions and cost-avoidance projections based on internal data can also enhance funding proposals.

Involving a finance representative and other key stakeholders (e.g., dieticians, nurses, and lactation consultants) in internal discussions may further increase understanding of the clinical and economic rationale for an EHMD and build internal advocacy for implementation. Funding barriers may be easier to overcome in the small number of states that allow Medicaid reimbursement for PDHM and HMB-HMF. Convincing the hospital administration to forego yearly re-justification of EHMD costs would also save time and increase program efficiency.

### Study limitations

The data reported here were collected at a roundtable meeting of experts and were not collected as part of a randomized, blinded, controlled clinical study; thus, the authors cannot rule out confounding factors that might be responsible for the morbidity rates and resulting cost-avoidance reported. It also cannot be ruled out that changes in other medical support not related to nutrition contributed to the differences in outcomes reported.

Another potential limitation of this work was the fact that all participating institutions used HMB-HMF manufactured by the same company (Prolacta Bioscience Inc., City of Industry, CA), which was the only commercially available HMB-HMF at the time these EHMD programs were implemented. Individual centers planning to implement an EHMD should evaluate the available products, and data supporting their use, in order to choose those that are most suitable for their particular needs.

## Conclusion

Although the clinical and economic impact of an EHMD in the NICU varied across institutions, benefits were consistently observed in terms of reduced complication rates (NEC, BPD, ROP, and late-onset sepsis), shorter LOS, and reduced costs (Figs. 1 and 2). A key strength of this analysis is the participation of institutions that differed widely in terms of size and patient populations, as well as their EHMD implementation strategies. Thus, the real-world data presented here support widespread adoption of an EHMD as a cost-effective approach for improving neonatal outcomes, complementing data from published cost-effectiveness analyses and clinical trials.^3–6,11,17,28^ Multicenter, prospective, real-world analyses are still needed to build on the information presented here in order to help pave the way for NICU leaders to make a persuasive case to hospital administrators for EHMD implementation to improve outcomes in very preterm infants.

## Data Availability

The datasets used and/or analyzed during the current study are available from the corresponding author on reasonable request.

## Abbreviations

BMB-HMFs: Bovine milk–based human milk fortifiers
BPD: Bronchopulmonary dysplasia
CLABSI: Central line-associated-blood stream infection
EHMD: Exclusive human milk diet
HMB-HMF: Human milk–based human milk fortifiers
LOS: Length of stay
MOM: Mother's own milk
NEC: Necrotizing enterocolitis
NICUs: Neonatal intensive care units
PDHM: Pasteurized donor human milk
ROP: Retinopathy of prematurity
VLBW: Very low birth weight
